# Hepatoprotective effects of aspirin on diethylnitrosamine-induced hepatocellular carcinoma in rats by reducing inflammation levels and PD-L1 expression

**DOI:** 10.1038/s41598-023-48812-z

**Published:** 2023-12-04

**Authors:** Yikai Wang, Muqi Wang, Chenrui Liu, Miao Hao, Wenjun Wang, Yaping Li, Juanjuan Shi, Xin Zhang, Shuangsuo Dang

**Affiliations:** Department of Infectious Diseases, The Second Affiliated Hospital of Xi’an Jiaotong University, No.157, Xiwu Road, Xi’an, 710004 Shaanxi China

**Keywords:** Liver cancer, Cancer models, Cancer prevention

## Abstract

Aspirin, as a widely used anti-inflammatory drug, has been shown to exert anti-cancer effects in a variety of cancers. PD-L1 is widely expressed in tumor cells and inhibits anti-tumor immunity. This study aims to clarify whether aspirin exerts its anti-hepatocellular carcinoma (HCC) effect by inhibiting PD-L1 expression. The rat model of HCC was established by drinking 0.01% diethylnitrosamine (DEN), and aspirin was given by gavage. The gross and blood biochemical indexes of rats were analyzed. CD4 and CD8 expression in liver tissues were investigated by immunohistochemistry. CCK8 assay was used to detect the inhibitory effect of aspirin on the proliferation of HCC cells. The regulatory effect of aspirin on PD-L1 expression was analyzed by western blot. As a result, the tumor number and liver weight ratio in the DEN + ASA group were lower than those in the DEN group (*P* = 0.006, *P* = 0.046). Compared with the DEN group, the expression of CD4 in the DEN + ASA group was significantly increased, while CD8 was decreased (all *P* < 0.01). Biochemical indexes showed that there were differences in all indexes between the DEN and control group (*P* < 0.05). The levels of DBIL, ALP, and TT in the DEN + ASA group were lower than those in the DEN group (*P* = 0.038, *P* = 0.042, *P* = 0.031). In the DEN group, there was an obvious fibrous capsule around the tumor, and the portal vein was dilated. The pathological changes were mild in the DEN + ASA group. Compared with the DEN group, the expression of PD-L1 in liver tissue of the DEN + ASA group was decreased (*P* = 0.0495). Cytological experiments further showed that aspirin could inhibit the proliferation and PD-L1 expression in Hep G2 and Hep 3B cells. In conclusion, aspirin can inhibit the proliferation of HCC cells and reduce tumor burden by reducing inflammation and targeting PD-L1.

## Introduction

Hepatocellular carcinoma (HCC) is a highly prevalent and malignant tumor worldwide, with over 840,000 new cases diagnosed annually^1^. It ranks sixth in terms of incidence and third in mortality^1^. Recent cancer statistics reveal a discouraging 5-year survival rate of approximately 15% for liver cancer, highlighting its aggressive nature^2^. The primary risk factors for HCC include viral infections such as hepatitis B virus (HBV) and hepatitis C virus (HCV), as well as chronic hepatitis and chronic alcoholism^3–5^. In China, the situation is further exacerbated by a higher prevalence of viral hepatitis infections. Many individuals with hepatitis B and C progress to liver cirrhosis, which can ultimately lead to the development of liver cancer^6, 7^. Annually, China witnesses over 466,000 new liver cancer cases and more than 422,000 deaths^8^. Sadly, HCC is frequently diagnosed at an advanced stage, resulting in a low rate of surgically removable cases, estimated to be only 10–15%^9^. Consequently, the identification of drugs for early prevention and the delay of liver cancer progression assumes critical significance.

Aspirin is a commonly used non-steroidal anti-inflammatory drug in clinical practice. Its main mechanism of action is to inhibit cyclooxygenase (COX) to exert anti-inflammatory and anti-platelet effects. Therefore, aspirin is often used for the prevention and treatment of cardiovascular and cerebrovascular diseases, as well as the prevention of postoperative thrombosis. As early as 1968, Gasic et al. found that tumor metastasis was also reduced in mice with thrombocytopenia^10^, and the inhibition of platelets by aspirin via inhibition of COX-1 seems a plausible explanation for its anticancer effect^11^. Therefore, exploring the anti-cancer mechanism of aspirin is of great significance for the clinical treatment of cancer in the future.

In recent years, immune checkpoint regulators such as Programmed Death Receptor 1 (PD-1)/Programmed cell death 1 ligand 1 (PD-L1) have emerged as effective targets for cancer therapy, garnering increasing attention^12^. PD-1, an immune checkpoint inhibitory receptor, is commonly expressed on immune cells and plays a crucial role in activating immunosuppressive signaling by binding to its ligand PD-L1^13^. PD-L1 is frequently expressed in tumor cells and contributes to their spread within the body. By binding to PD-1 on T cells, highly expressed PD-L1 allows cancer cells to evade immune cell recognition, facilitating their metastasis^14^. Research has indicated that aspirin can hinder the progression of ovarian^15^, lung^16^, and colorectal^17^ cancer by inhibiting PD-L1 expression. However, the role of PD-L1 in aspirin inhibition of HCC remains unclear.

Liver function indicators, such as bilirubin, ALT, AST, albumin (ALB), and related markers, crucially reflect liver health and are closely associated with HCC development. Bilirubin, categorized into direct and indirect forms, undergoes breakdown and elimination within the liver. HCC frequently leads to bilirubin accumulation and subsequent jaundice due to liver cell damage. Research indicates that bilirubin, particularly in combination with PIVKA-II and AFP, serves as a diagnostic marker for HCC^18^. Transaminases are closely related to liver inflammation, and high levels of ALT and AST increase the risk of HCC, especially in males and patients with viral hepatitis^19, 20^. Total protein (TP) and its constituent, ALB, significantly impact HCC prognosis, with studies indicating that low TP and ALB levels correlate with shorter survival in HCC patients^21, 22^. Elevated alkaline phosphatase (ALP) levels, commonly observed in cholestasis and hepatocyte damage, serve as an independent risk factor for HCC patient prognosis^23^. Furthermore, cholinesterase (CHE), a primary marker of hepatic protein synthesis, has been reported as a crucial predictor of prognosis in HCC patients undergoing sorafenib treatment^24^. Cholesterol (TCHO) and triglyceride (TG) levels similarly reflect the inflammatory state of the liver, and elevated levels are associated with increased HCC risk^25, 26^.

Platelet (PLT) counts and coagulation indicators, including fibrinogen (FIB), prothrombin time (PT), activated partial thromboplastin time (APTT), among others, frequently exhibit abnormalities in HCC. Previous studies have shown that preoperative low levels of PLT indicate poor prognosis in HCC^27^. Elevated levels of FIB and PT among coagulation markers have been linked to a poorer prognosis^28, 29^. Moreover, APTT serves as an independent prognostic risk factor for early HCC recurrence within 1 year following curative hepatectomy^30^. Hence, early intervention and detection of PLT and coagulation indicators are particularly important for predicting the progression and prognosis of HCC.

Therefore, this study aims to establish a rat model of liver cancer and intervene with aspirin, analyze the changes in gross indexes, blood biochemical indexes, coagulation indexes, and T cell count in rats, and explore the effect of aspirin on the progression of liver cancer. Furthermore, the expression of PD-L1 in liver tissues and liver cancer cell lines under different intervention conditions was compared to explore the potential role of PD-L1 as a target in the inhibition of liver cancer by aspirin.

## Results

### Aspirin significantly inhibited the development and progression of DEN-induced liver cancer in rats

**Figure 1:**
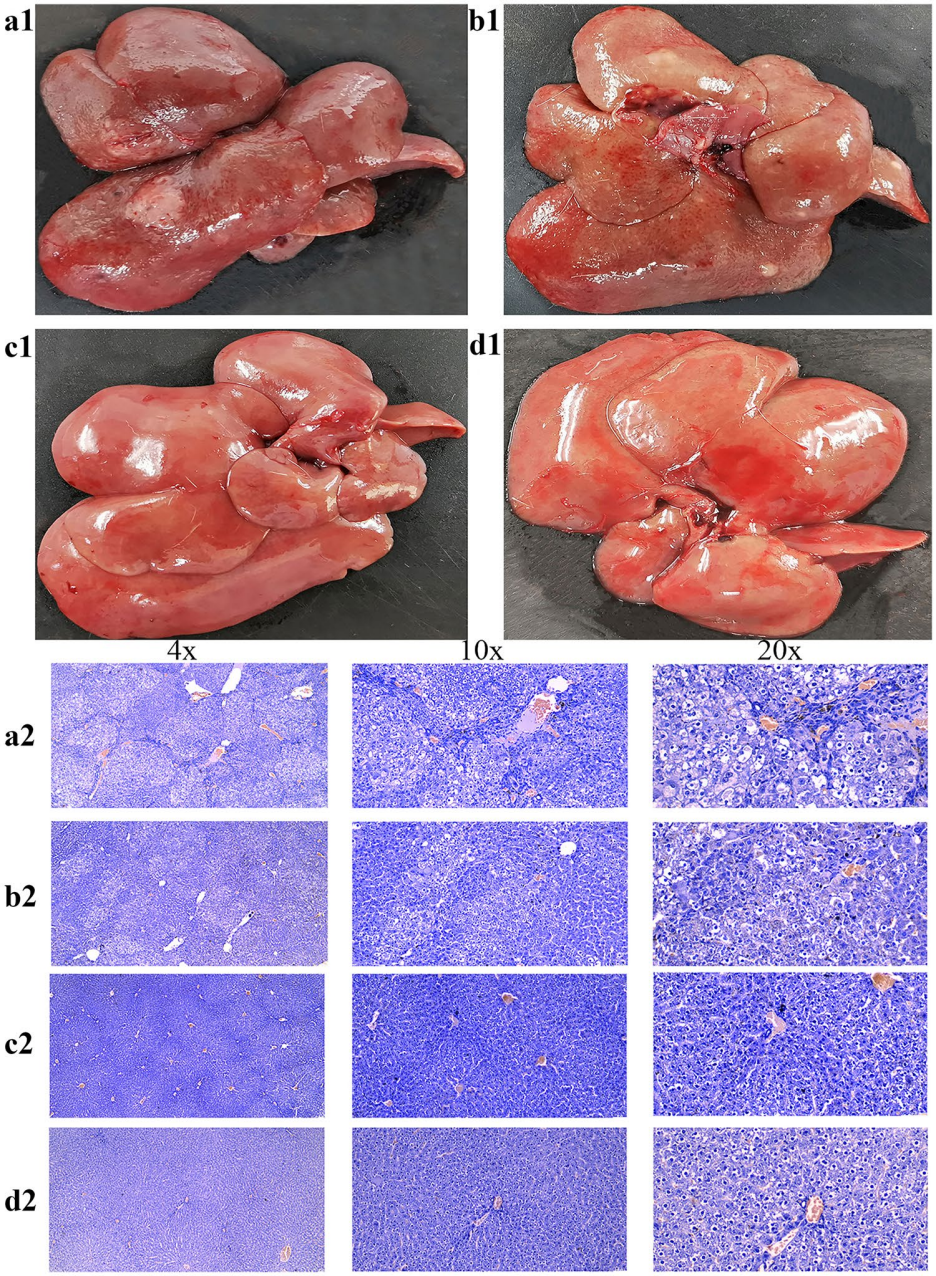
Gross observation and HE staining light of rat livers in each group: (**a1**,**a2**) Gross liver specimens and HE staining pictures of rats in the DEN group. There were gray-white tumor nodules on the liver surface, and HE staining showed cancer cell infiltration and pseudolobular expansion. (**b1**,**b2**) Gross liver specimens and HE staining pictures of rats in the DEN + ASA group. There were a few gray-white tumor nodules on the surface of the liver. HE staining showed a small amount of fibrous cysts around the cancer and the structure of liver lobules was clear. (**c1**,**c2**) Gross liver specimens and HE-stained pictures of rats in the control group. The surface of the liver was smooth, and HE staining showed that the morphology of hepatocytes was normal and the structure of hepatic lobules was clear. (**d1**,**d2**) Gross liver specimens and HE staining pictures of rats in the ASA group. The surface of the liver was smooth, and HE staining showed clear hepatic lobules without inflammatory cell infiltration.

The observation of the gross liver specimens showed that: all 10 rats in the DEN group had several gray-white tumor nodules of varying sizes on the surface of the liver, some of which had hemorrhage and necrosis; the liver outside the tumor nodules was rough, and tough (Fig. 1a1); the DEN + ASA group there were several gray-white tumor nodules of varying sizes on the liver surface of 8 rats, and the other 2 had no tumor nodules visible to the naked eye, and the liver surface outside the tumor nodules was smooth and tough (Fig. 1b1). The livers of the ASA group and the control group were smooth, ruddy in color, and soft (Fig. 1c1,d1).

The cancer nodule specimens of 18 rats were observed by HE staining, all of which were HCC. Most of the cancer cells were arranged in a mass, and infiltration into the surrounding tissues could be observed, and some areas had hemorrhage and necrosis. In the DEN group, the peritumoral fibrous capsule was significantly thickened, and the tissues adjacent to the cancer nodules formed obvious pseudolobules; dilated portal veins and hyperplastic bile ducts were seen in the liver tissue, and a large number of fibrous connective tissue hyperplasia extended into the lobules (Fig. 1a2). Compared with the DEN group, the fibrous capsule around the cancer was significantly reduced in the DEN + ASA group; the morphology of the hepatocytes was normal, the lobular structure was clearer, and there was less inflammatory cell infiltration (Fig. 1b2). In the control and ASA groups, the morphology of hepatocytes was normal, the structure of the hepatic lobule was clear, and no inflammatory cell infiltration was observed (Fig. 1c2,d2).

**Figure 2:**
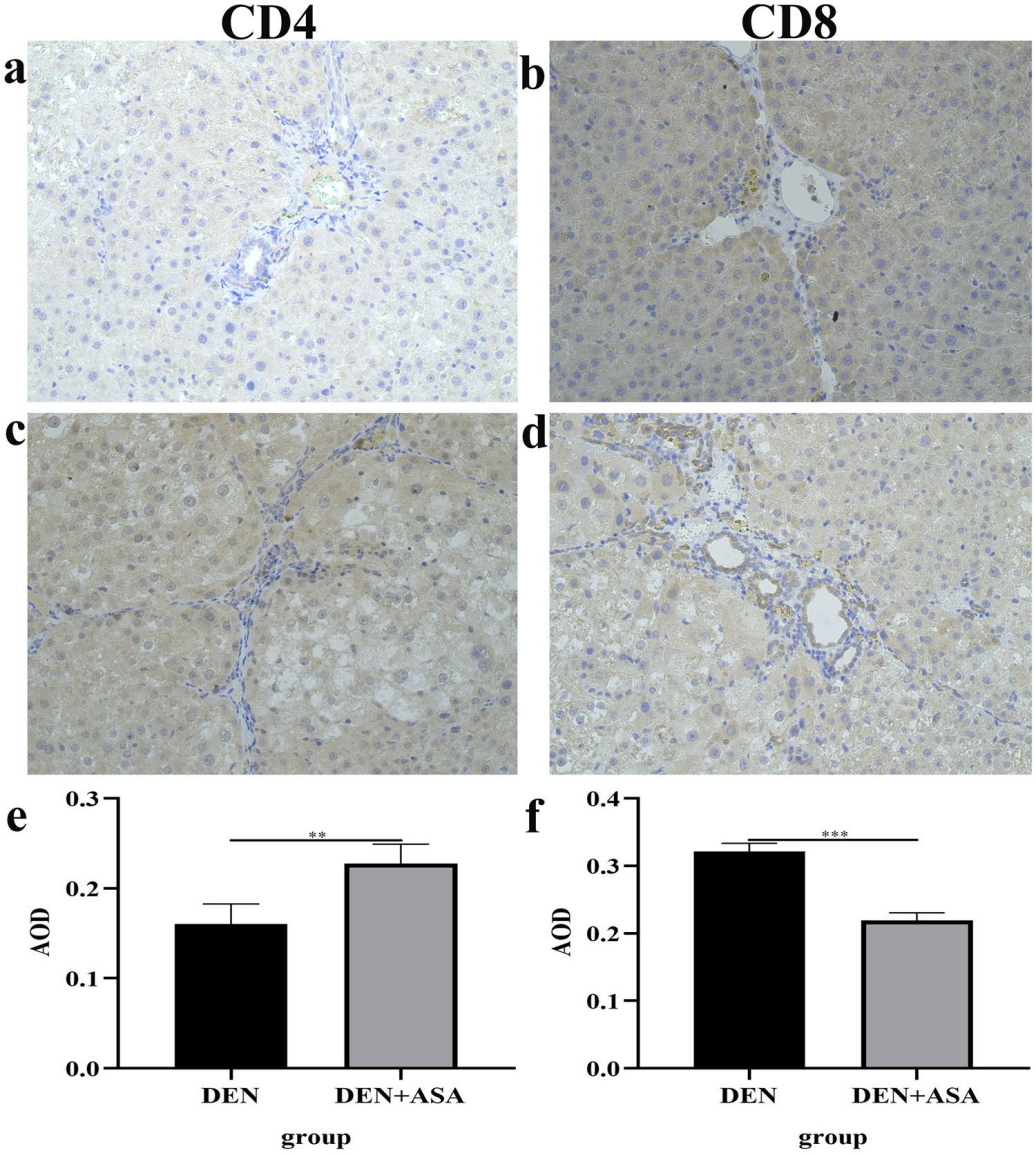
IHC staining of CD4 and CD8 in liver tissues of rats in different groups: (**a**,**b**) Expression of CD4 and CD8 in the DEN group was detected by IHC. (**c,d**) Expression of CD4 and CD8 in the DEN + ASA group was detected by IHC. (**e**) The expression of CD4 in the DEN + ASA group was significantly higher than that in the DEN group (*P* < 0.01). (**f**) The expression of CD8 in the DEN + ASA group was significantly lower than that in the DEN group (*P* < 0.001). The images for IHC staining were all at 20X magnification. **P < 0.01; ***P < 0.001.

To further explore the effect of aspirin use on immune cells, we used IHC staining to analyze the expression of CD4 and CD8 in the DEN group and DEN + ASA group. Semi-quantitative analysis revealed that the AOD of CD4 was significantly higher in the DEN + ASA group than in the DEN group (*P* = 0.0012) (Fig. 2a,c). Interestingly, however, the AOD of CD8 was significantly lower in the DEN + ASA group than in the DEN group (*P* < 0.001) (Fig. 2b,d).

### Aspirin significantly reduces the tumor burden of DEN-induced liver cancer in rats

**Figure 3:**
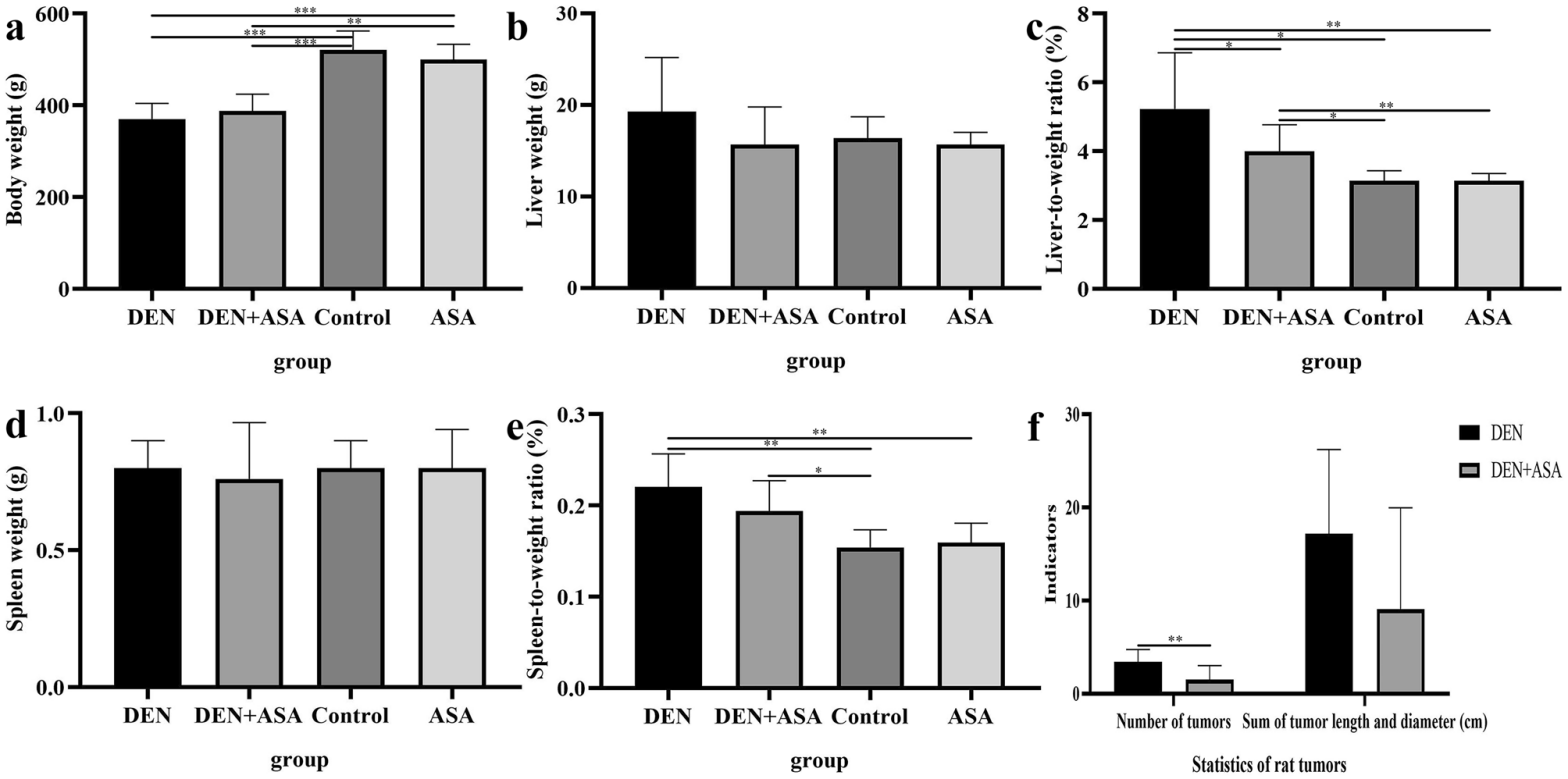
Tumor burden of rats in each group: The rats in the DEN group, the DEN + ASA group, the control group, and the ASA group were compared in terms of (**a**) body weight, (**b**) liver weight, (**c**) liver-to-weight ratio, (**d**) spleen weight and (**e**) spleen-to-weight ratio. (**f**) The number of tumors and the sum of tumor length and diameter were compared between the DEN group and the DEN + ASA group. *P < 0.05; **P < 0.01; ***P < 0.001.

By measuring the weight, liver weight, and spleen weight of rats, it was found that the weight of the DEN group was significantly decreased compared with the control group and ASA group (all *P* < 0.001) (Fig. 3a); the liver body weight of the DEN group was significantly increased compared with that of the control group (*P* = 0.016), ASA group (*P* = 0.002) and DEN + ASA group (*P* = 0.046) (Fig. 3c); the spleen body weight ratio was significantly increased compared with that of the control group (*P* = 0.004) and ASA group (*P* = 0.004) (Fig. 3e). In addition, we also found that compared with the control group (*P* = 0.003) and ASA group (*P* = 0.007), the body weight of rats in the DEN + ASA group was significantly decreased, while the liver weight/body weight ratio was significantly increased (*P* = 0.032; *P* = 0.007) (Fig. 3a,c). Similarly, the spleen/body weight ratio of the DEN + ASA group was significantly higher than that of the control group (*P* = 0.028) (Fig. 3e). There were no significant differences in body weight, liver weight, spleen weight, liver-to-weight ratio, and the spleen-to-weight ratio between the ASA group and the control group (all *P* > 0.05) (Fig. 3). Combined with the tumor situation, it showed that the DEN group had a higher tumor burden, and the use of aspirin could effectively reduce the tumor burden.

Further, we counted tumor nodules with a diameter of more than 2 mm and analyzed the tumor formation, tumor number, and tumor length and diameter in each group. The analysis found that aspirin intervention significantly reduced the number of tumors (*P* = 0.006). In addition, the sum of the tumor long diameter in the intervention group was also smaller than that in the model group (9.1 vs. 17.2), but the difference was not statistically significant (*P* = 0.074) (Fig. 3f).

### Aspirin significantly reduces serum DBIL and ALP levels in DEN-induced liver cancer rats

**Figure 4:**
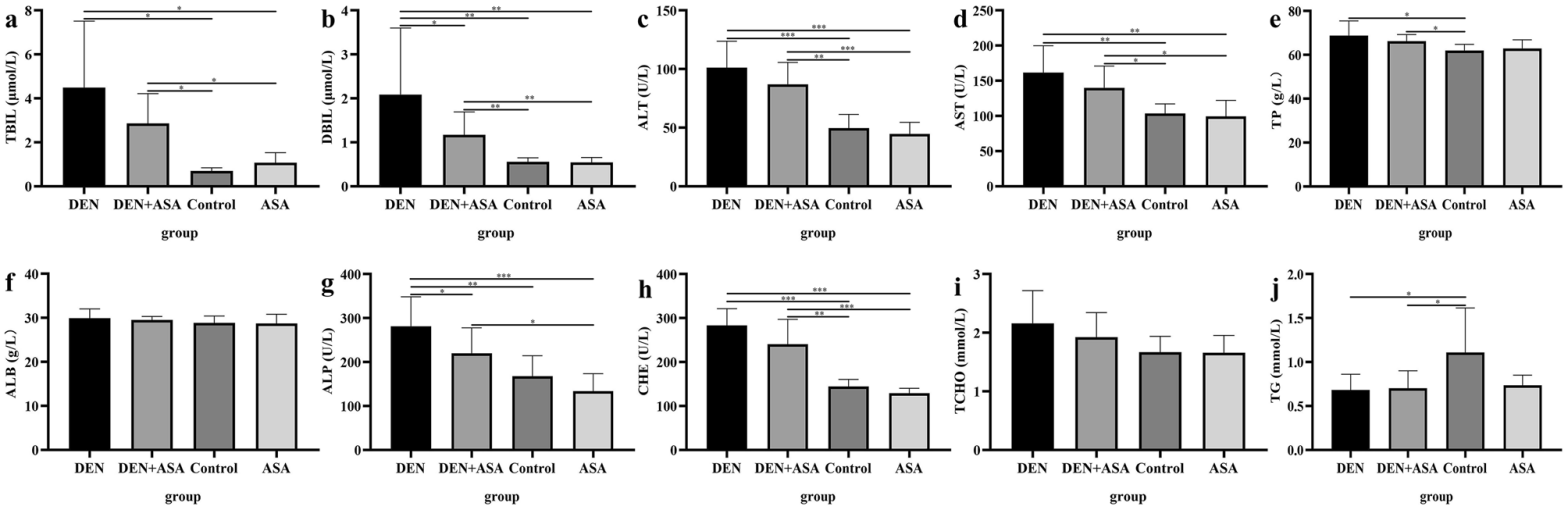
Biochemical changes in each group: The biochemical index (**a**) TBIL, (**b**) DBIL, (**c**) ALT, (**d**) AST, (**e**) TP, (**f**) ALB, (**g**) ALP, (**h**) CHE, (**i**) TCHO, and (**j**) TG of rats in DEN group, DEN + ASA group, control group, and ASA group was compared. *P < 0.05; **P < 0.01; ***P < 0.001.

Compared with the control group and ASA group, the levels of total bilirubin (TBIL), direct bilirubin (DBIL), alanine aminotransferase (ALT), aspartate aminotransferase (AST), and CHE in DEN group and DEN + ASA group were significantly increased (all* P* < 0.05) (Figs. 4h, 4a–d). In addition, the TP in the DEN group and DEN + ASA group was significantly higher than that in the control group (*P* = 0.048, *P* = 0.024) (Fig. 4e). Similarly, ALP in the DEN group was significantly higher than that in the control and ASA groups (*P* = 0.005, *P* < 0.001), and the ALP level in the DEN + ASA group was also higher than that in the ASA group (*P* = 0.011) (Fig. 4g). However, TG levels in the DEN and DEN + ASA groups were significantly lower than those in the control group (*P* = 0.029, *P* = 0.040) (Fig. 4j). In addition, ALB and TCHO did not show significant differences among the groups (Fig. 4f,I).

By comparing the biochemical indexes of the DEN group and the DEN + ASA group, we found that the DBIL and ALP levels of the ASA intervention group were significantly lower than those of the model group (*P* = 0.038, *P* = 0.042) (Fig. 4b,g). In addition, there was no significant difference between the control group and the ASA group (all *P* > 0.05).

### Aspirin intervention can affect coagulation indexes in rats with liver cancer

**Figure 5:**
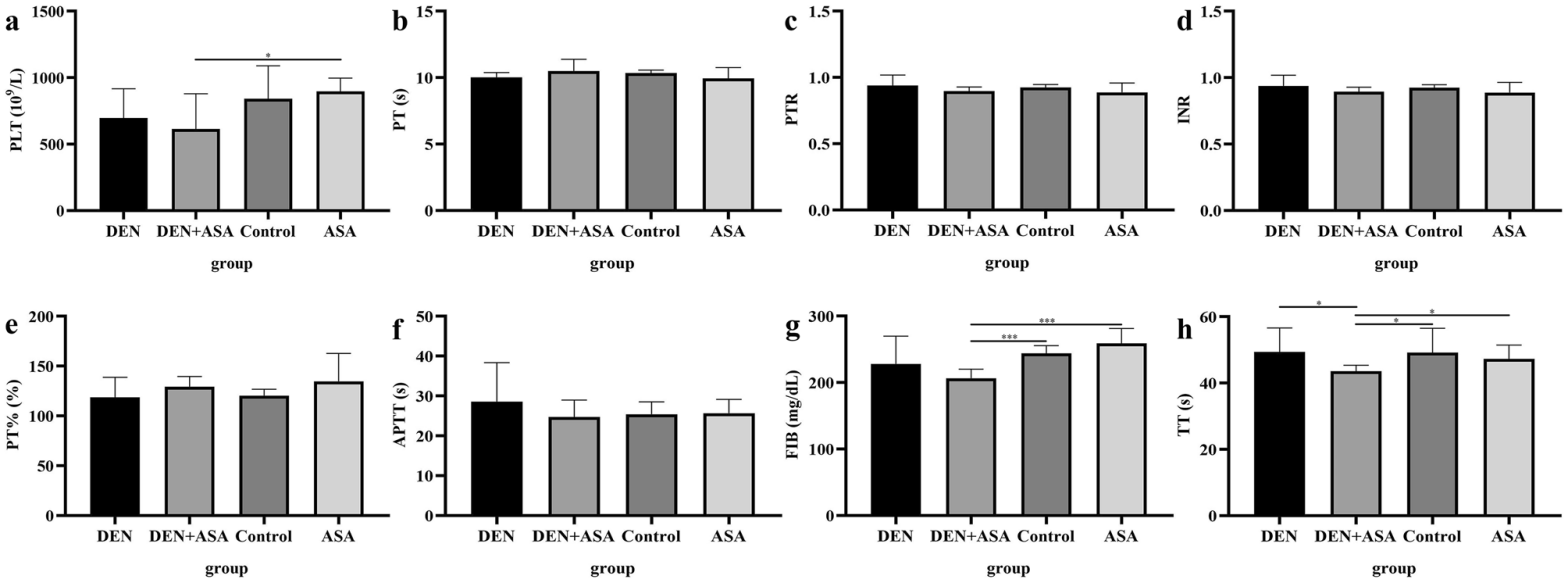
Changes in hematological indexes in each group: The hematological index (**a**) PLT, (**b**) PT, (**c**) PTR, (**d**) INR, (**e**) PT%, (**f**) APTT, (**g**) FIB, and (**h**) TT of rats in the DEN group, DEN + ASA group, control group, and ASA group were compared. *P < 0.05; ***P < 0.001.

Analysis of hematological indexes showed that there was no significant difference between the DEN group and the control group and the ASA group (*P* > 0.05). Interestingly, the PLT level in the DEN + ASA group was significantly lower than that in the ASA group (*P* = 0.046) (Fig. 5a); FIB and thrombin time measurements (TT) were significantly lower than those in the control (*P* < 0.001, *P* = 0.046) and ASA groups (*P* < 0.001, *P* = 0.038) (Fig. 5g,h). Compared with the DEN group, the TT in the DEN + ASA group was also significantly shortened (*P* = 0.031) (Fig. 5h). However, PT, prothrombin time ratio (PTR), international normalized ratio (INR), prothrombin activation (PT%), and APTT were not significantly different between the DEN group and the DEN + ASA group (Fig. 5b–f). In addition, there was no significant difference between the control group and the ASA group (all *P* > 0.05).

### Aspirin can inhibit the proliferation of liver cancer cell lines

**Figure 6:**
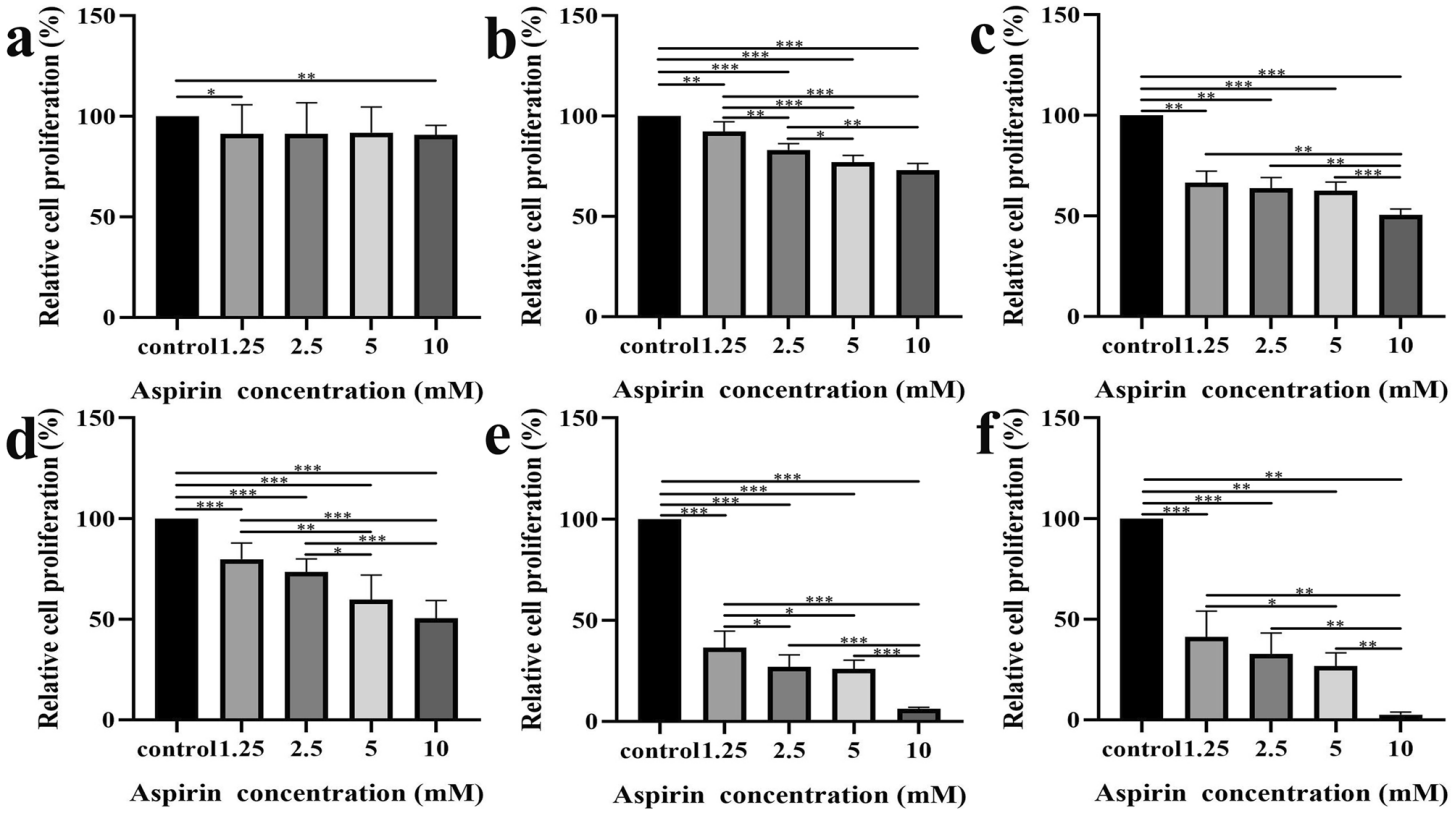
Inhibitory effect of aspirin on Hep G2 cell proliferation at different concentrations and time gradients: (**a**) The proliferation of Hep G2 cells treated with different concentrations of aspirin for 6 h was different from that of the control group. (**b**) The proliferation of Hep G2 cells treated with different concentrations of aspirin for 12 h was different from that of the control group. (**C**) The proliferation of Hep G2 cells treated with different concentrations of aspirin for 18 h was different from that of the control group. (**d**) The proliferation of Hep G2 cells treated with different concentrations of aspirin for 24 h was different from that of the control group. (**e**) The proliferation of Hep G2 cells treated with different concentrations of aspirin for 48 h was different from that of the control group. (**f**) The proliferation of Hep G2 cells treated with different concentrations of aspirin for 72 h was different from that of the control group. The concentration gradients of aspirin were set to 1.25, 2.5, 5, and 10 mM. *P < 0.05; **P < 0.01; ***P < 0.001.

**Figure 7:**
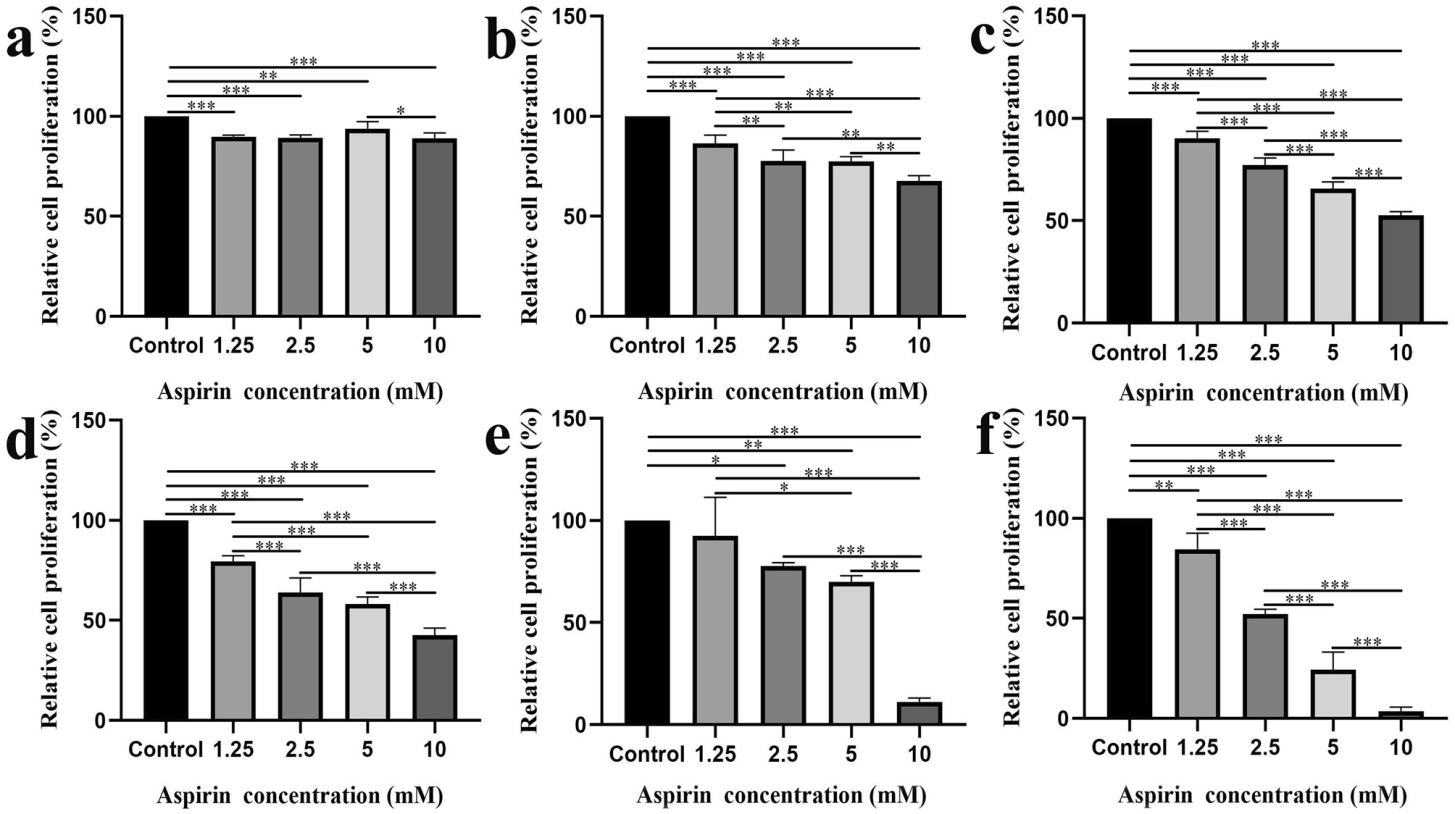
Inhibitory effect of aspirin on Hep 3B cell proliferation at different concentrations and time gradients: (**a**) The proliferation of Hep 3B cells treated with different concentrations of aspirin for 6 h was different from that of the control group. (**b**) The proliferation of Hep 3B cells treated with different concentrations of aspirin for 12 h was different from that of the control group. (**c**) The proliferation of Hep 3B cells treated with different concentrations of aspirin for 18 h was different from that of the control group. (**d**) The proliferation of Hep 3B cells treated with different concentrations of aspirin for 24 h was different from that of the control group. (**e**) The proliferation of Hep 3B cells treated with different concentrations of aspirin for 48 h was different from that of the control group. (**f**) The proliferation of Hep 3B cells treated with different concentrations of aspirin for 72 h was different from that of the control group. The concentration gradients of aspirin were set to 1.25, 2.5, 5, and 10 mM. *P < 0.05; **P < 0.01; ***P < 0.001.

We conducted experiments with different concentrations and durations of intervention to investigate the impact of aspirin on the growth of liver cancer cell lines under various conditions. Except for the 6-h intervention group, the proliferation of Hep G2 cells in the remaining five groups was significantly suppressed as the intervention time and aspirin concentration increased (compared to the control group, all *P* < 0.01) (Fig. 6). Furthermore, aspirin treatment was administered to Hep 3B cells to further confirm its inhibitory effect on liver cancer cell proliferation. As depicted in Fig. 7, the proliferation of Hep 3B cells in each group was significantly inhibited with the increase of intervention time and aspirin concentration (compared to the control group, all *P* < 0.05).

### Aspirin can inhibit the expression of PD-L1

**Figure 8:**
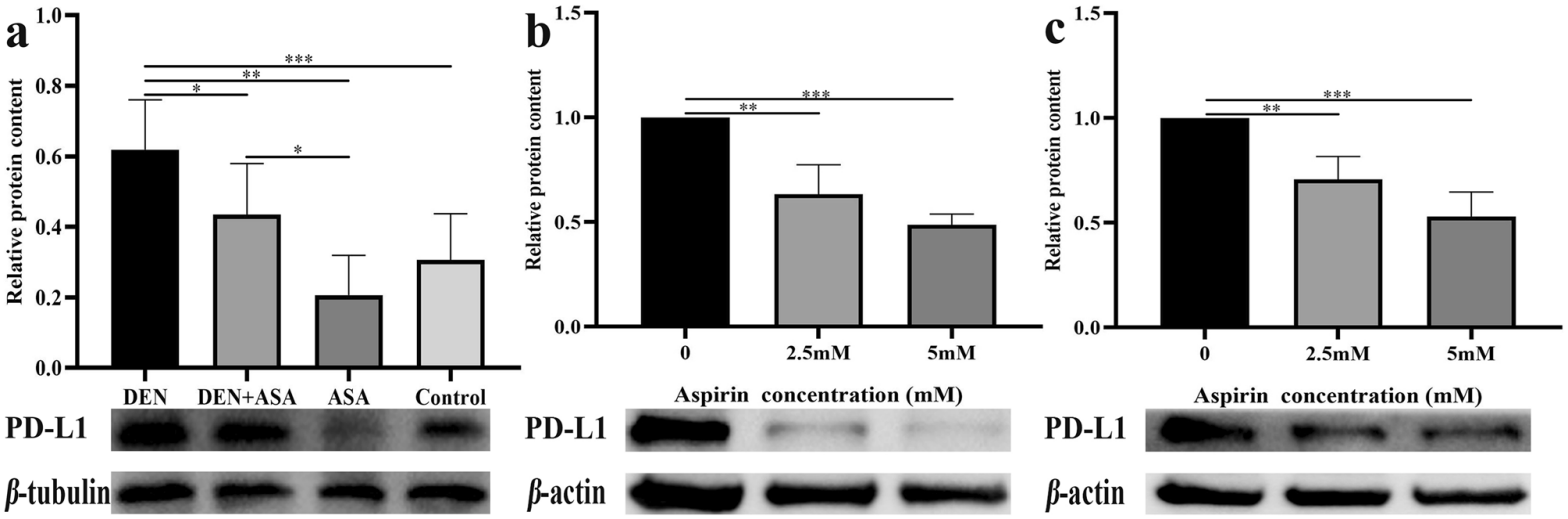
Aspirin significantly reduced the expression of PD-L1: (**a**) Differences in PD-L1 expression in liver tissues of rats in the DEN group, DEN + ASA group, ASA group, and control group. (**b**) The difference in PD-L1 expression in Hep G2 cells under different intervention concentrations compared with the control group. (**c**) The difference in PD-L1 expression in Hep 3B cells under different intervention concentrations compared with the control group. The blots were cut prior to hybridization with antibodies during blotting, and images of all replicate blots were included in Supplementary Fig. 1. *P < 0.05; **P < 0.01; ***P < 0.001.

To further clarify the mechanism of aspirin inhibiting the development of liver cancer, we detected the expression of PD-L1 in different groups of liver tissues. The expression of PD-L1 in the DEN group was significantly higher than that in the ASA group (*P* = 0.0002) and the control group (*P* = 0.0026). And by comparing the DEN group with the DEN + ASA group, it can be shown that aspirin can significantly inhibit the expression of PD-L1 (*P* = 0.0495) (Fig. 8a).

We further treated Hep G2 cells and Hep 3B cells with 2.5 mM and 5 mM aspirin for 24 h and analyzed PD-L1 expression. Figure 8b illustrates that the expression of PD-L1 was significantly lower in the 2.5 mM intervention group and the 5 mM intervention group compared to the control group (*P* = 0.0048; *P* = 0.0008). Similar results were observed when Hep 3B cells were treated with aspirin, as shown in Fig. 8c. The expression of PD-L1 in the 2.5 mM intervention group and the 5 mM intervention group was significantly lower than that in the control group as well (*P* = 0.0040; *P* = 0.0001).

## Discussion

Our study preliminarily demonstrated that aspirin could inhibit the occurrence of HCC by reducing the expression of PD-L1, and this study is the first according to our literature review.

HCC is one of the top three cancers causing the most deaths, and the survival and prognosis of HCC patients are poor due to delayed diagnosis and lack of effective treatment strategies. Undoubtedly, there is an urgent need for an efficient and low-toxic treatment method to prolong the overall survival time of HCC patients. Numerous previous studies have shown that aspirin plays an important role in cancer prevention and treatment. Our previous meta-analysis including large population studies also demonstrated that aspirin can inhibit the occurrence and progression of liver cancer^31^. In this experiment, we confirmed that aspirin can significantly inhibit the occurrence of liver cancer (*P* = 0.006) and reduce the tumor burden (*P* = 0.046). PD-L1, as an important immune checkpoint molecule and involved in weakening the immune response to infection, can allow cancer cells to escape immune surveillance^32^ and has been shown in ovarian cancer, melanoma, colon adenocarcinoma, lung squamous cell carcinoma, breast adenocarcinoma, and many other cancers^33^. However, whether PD-L1 plays a role in aspirin inhibition of HCC remains unclear. In this experiment, we found that the level of PD-L1 in the DEN + ASA group was significantly lower than that in the DEN group (*P* = 0.0495). This result proves that aspirin can significantly reduce the expression of PD-L1 at the protein level in cancer cells, so PD-L1 is a new target for aspirin to inhibit the growth of liver cancer. Interestingly, we found that there was no significant difference in PD-L1 expression between the Control group and the ASA group (*P* = 0.1901). Therefore, we speculate that because PD-L1 is abundantly expressed in cancer cells, it leads to tumor immune escape, while normal cells express little or no PD-L1. Therefore, the inhibitory effect of aspirin is mainly reflected in cancer cells and has less effect on normal cells. Moreover, our in vitro experiments on two HCC cell lines additionally showcased that aspirin effectively suppressed the growth of HCC cells by inhibiting the expression of PD-L1.

Although our study demonstrated that aspirin can reduce the expression of PD-L1 in HCC, the specific signaling pathway of aspirin action remains unclear. In the study of other cancers, Zhang et al. found that aspirin could inhibit the growth of lung cancer cells by targeting the TAZ/PD-L1 axis^16^. In addition, Xiao et al.’s study of ovarian cancer found that aspirin inhibited the expression of PD-L1 by inhibiting KAT5, thereby inhibiting the signaling pathway of PD-1 and PD-L1 to attenuate the progression of ovarian cancer^15^. Henry et al. showed that aspirin inhibited the growth of PI3K-mutant breast cancer by activating AMP-activated protein kinase (AMPK) and inhibiting the mammalian target of rapamycin complex 1 (mTORC1), independent of its effects on cyclooxygenase-2 (COX-2) and nuclear factor-kappa B (NF-κB)^34^. The above studies suggest that aspirin may act on multiple targets in HCC to suppress PD-L1 expression by regulating an integrated cellular signaling network. In addition, we note a study by Zuazo et al. demonstrating that PD-L1/PD-1 blockade induces the expansion of systemic CD8 + and CD4 + T cell subsets to exert a direct antitumor response^35^. Our study also showed that the use of aspirin can upregulate CD4 expression and inhibit PD-L1 expression, which coincides to some extent with their study. However, interestingly, our study found that the use of aspirin simultaneously inhibited CD8 expression (Fig. 2b,d). The reason for this contradictory result may be that most of the previous studies used specific inhibitors of PD1/PD-L1, such as nivolumab and atezolizumab. However, aspirin has a wide range of action pathways, and the specific mechanism by which aspirin inhibits the expression of PD-L1 in liver cancer cells is still unclear. Therefore, aspirin may act on multiple signaling pathways and ultimately suppress CD8 expression.

It is well known that inflammation is closely related to the development of tumors. In the inflammatory cascade in which inflammatory cells provide the basis for the development of the tumor microenvironment, disrupting this cascade may prevent further proliferation of malignant cells^36^. In this experiment, the DEN + ASA group exhibited a lower presence of fibrous capsules surrounding cancer and no noticeable infiltration of inflammatory cells under microscopic examination. These findings suggest that aspirin can potentially impede liver cancer progression by suppressing inflammation levels, minimizing the incidence of liver cancer, and alleviating the tumor burden. Our research further demonstrated that aspirin had a significant impact on reducing DBIL and ALP levels in HCC rats, as indicated in Fig. 4 of our blood biochemical analysis. It is well known that DBIL is converted from IBIL by UDP-glucuronosyltransferase 1A1, and both together form TBIL^37^. Previous studies have found that increased DBIL often indicates hepatocyte injury^38^. In a retrospective study of NAFLD patients, Salomone et al. found that unconjugated bilirubin levels were lower in patients with high degrees of liver inflammation and fibrosis, indicating more conversion of IBIL to DBIL^39^. Therefore, DBIL levels are closely related to the degree of liver inflammation. The significant decrease in DBIL level in the aspirin intervention group compared with the DEN group also suggested that the use of aspirin reduced the level of liver inflammation, thereby inhibiting the progression of HCC. ALP, a hydrolytic enzyme highly expressed in the liver, is associated with poor prognosis in HCC^40, 41^. The abnormal increase of ALP is usually caused by cholestasis and liver inflammation, which can also lead to the development of HCC. In our study, the use of aspirin significantly reduced ALP expression levels in HCC rats compared with the DEN group, which should also be achieved by the anti-inflammatory effect of aspirin. Additionally, we observed significantly elevated levels of TBIL, ALT, AST, and CHE in both the DEN and DEN + ASA groups compared to the control group. However, there was no notable difference between the two treatment groups (Fig. 4). As conventionally understood, TBIL, ALT, AST, and CHE are usually elevated in response to liver inflammation^42, 43^. Consequently, all these indices were significantly higher in the DEN group than in the control group. Intriguingly, ASA treatment exhibited a tendency to mitigate liver inflammation and subsequently reduce these parameters, albeit without significant differences observed. TP and its major component, ALB, are mainly synthesized by the liver, and therefore liver cancer is often reduced^44^. However, in our study, TP was higher in the DEN group and the DEN + ASA group than in the control group, which may be due to the fact that the chronic wasting stage of HCC has not yet been entered. TCHO metabolism is closely related to the liver, and liver function disorders due to HCC affect TCHO metabolism. Our study revealed a slight elevation in TCHO levels in rats from both the DEN and DEN + ASA groups, although the difference was not statistically significant (Fig. 4i). TG is mainly synthesized by the liver, and previous studies have shown that TG reduction is closely related to the high risk of HCC^45^. A population study in Korea also showed that decreased TG levels increased the occurrence of HBV-related liver cancer^46^. In our study, TG levels in the DEN and DEN + ASA groups were significantly lower than those in the control group (Fig. 4j), suggesting that low TG levels are highly correlated with HCC, which coincides with the results of the above two population studies.

However, our study also indicates a potential risk of bleeding with aspirin use. Given that the liver is the main site for the synthesis of coagulation factors, both exogenous and endogenous coagulation pathways are highly dependent on the liver^47^. Consequently, alterations in the quantity and quality of coagulation factors due to liver disease result in varying degrees of coagulation dysfunction. However, aspirin's anti-platelet and coagulation effects^48^ might potentially heighten the bleeding risk among HCC patients. Our study demonstrated this risk, as indicated in Fig. 5, where PLT, FIB, and TT indexes in the DEN + ASA group were lower compared to the control or ASA groups. It is worth noting that although PT, PTR, INR, PT%, and APTT were not significantly different between groups, we still cannot ignore the potential risk of bleeding (Fig. 5b–f). Because a variety of cytokines are involved in the balance of hemostasis, PT and INR will not be sufficient to show the true state of the body when there is a lack of procoagulant and anticoagulant factors at the same time^47^.

In conclusion, our findings identify a novel mechanism by which aspirin alleviates liver cancer. Aspirin has been shown to fight the growth of liver cancer. PD-L1 has been determined to be decreased in aspirin-suppressed liver cancer. Aspirin inhibits the expression of PD-L1 and causes liver cancer growth arrest. In addition, the anti-inflammatory effect of aspirin has also enhanced its effect of blocking the occurrence and development of liver cancer to a certain extent. Our findings suggest that aspirin may be a promising new drug for liver cancer treatment. In the future, the upstream molecular mechanism of its inhibition of PD-L1 expression in liver cancer cells should be explored in more detail, and a large-scale population study should be conducted to explore the advantages and disadvantages of its single drug and combined targeted drugs in the treatment of liver cancer.

## Methods

### Establishment of animal liver cancer model and specimen processing

Thirty male SD rats (purchased from the Experimental Animal Center of Xi’an Jiaotong University), weighing about 170–210 g, were reared in separate cages, fed with standard chow, regularly changed bedding, and fed ad libitum for one week. From the second week, the rats were randomly divided into the control group (n = 5), ASA group (n = 5), DEN group (n = 10), and DEN + ASA group (n = 10). DEN group and DEN + ASA group were prepared with distilled water to prepare a 0.01% DEN (McLean, 99.9% purity) solution. 0.01%DEN was freely consumed for 6 weeks, followed by 1 week of discontinuation, followed by DEN feeding for 10 weeks and discontinuation^49^. The control group was given free drinking water; the ASA group and the DEN + ASA group were given aspirin (10 mg/kg per day) by gavage. The general conditions of the animal were observed every day^50^. Rats can be fed in individual cages if they are in very poor condition.

Before euthanasia, all animals were anesthetized with sodium pentobarbital (30 mg/kg, intraperitoneal injection). After successful anesthesia, blood was taken and the liver and spleen were obtained. Then, the relevant data such as liver size, color, texture, weight, and presence or absence of cancer nodules were rapidly observed and recorded. Finally, liver tissues were fixed in 4% paraformaldehyde solution for 24–48 h, embedded in paraffin, and cut into 3–4 μm thick sections for HE staining.

The experimental procedures were approved by the Medical Ethics Committee of the Second Affiliated Hospital of Xi’an Jiaotong University. The study was reported in accordance with the ARRIVE guidelines. All procedures were carried out in accordance with institutional guidelines.

### Immunohistochemical analyses

Rat liver sections with a thickness of 5 μm were cut from paraffin blocks and mounted on slides. Deparaffinized hydration was performed using xylene and varying concentration gradients of alcohol. Then, antigen thermal repair was performed using a citric acid antigen repair solution at pH 6.0. After antigen repair was completed, endogenous peroxidase was blocked using 3%H_2_O_2_ and 10% goat serum. Further, rabbit polyclonal antibodies against CD4 (Servicebio, China, dilution: 1:200) and CD8 (Servicebio, China, dilution: 1:500) were dropped onto each section and incubated overnight at 4 °C. The second antibody (goat anti-rabbit, Abcam, USA) was incubated the following day and stained with DAB. Image-J software (NIH, USA) was used for analysis. The intensity of CD4 and CD8 positive staining was expressed as the mean optical density value (AOD) and calculated using the formula: AOD = integrated optical density (IOD)/area^51^.

### Cell lines

Hep G2 (HCC; Cat. No. CL-0103) and Hep 3B (HCC; Cat. No. CL-0102) cell lines were purchased from Procell Life Science & Technology. We used Procell Life Science & Technology’s DMEM high glucose complete medium which was supplemented with 10% FBS (164210-50), 1% P/S (PB180120), and 1% GlutaMax (PB180419). Cells were incubated at 37 °C and 5% CO_2_.

### Cell proliferation analysis

Cell proliferation was detected by CCK8 assay (Glpbio, USA). Cells were seeded in 96-well plates at a density of 3000 cells per well in at least three replicates. The cells were incubated for 24 h at 37 °C in an incubator with 5%CO_2_. The medium containing aspirin was changed and different ASA concentrations were set: 1.25 mM, 2.5 mM, 5 mM, and 10 mM^52^. The cells were cultured for 6, 12, 18, 24, 48, and 72 h^53^. It was then added to each well at a ratio of 10μL CCK8 to 90 μl medium, incubated for 4 h at OD 450 nm, and the absorbance value was measured by an absorbance meter.

### Western blot

Cells and liver tissues from different aspirin-treated groups were lysed using RIPA buffer (Servicebio, China). Protein concentrations were determined using the BCA Protein Assay kit (Servicebio, China), and protein samples (20 μg/well) were separated by 10% SDS-PAGE and transferred to PVDF membranes. The cells were blocked with 5% low-fat milk for 2 h and incubated with primary antibodies overnight at 4 °C. Secondary antibodies were incubated the next day and analyzed by ChemiDoc MP Imaging System (Bio-Rad, USA). The primary antibodies used in this study, are anti-PD-L1 (GeneTex, USA), anti-β-actin (Abcam, USA), and anti-β-tubulin (Wanleibio, China). The secondary antibodies included goat anti-rabbit (Abcam, USA) or anti-mouse antibody (Abcam, USA). The blots were cut prior to hybridization with antibodies during blotting, and images of all replicate blots were included in Supplementary Fig. 1.

### Statistical analysis

Each experiment was performed at least three times. SPSS 26.0 statistical software was used for data processing, and measurement data were expressed as ${\overline{\text{x}}}$ ± s. The non-parametric test in the SPSS26.0 software package was used to test the normality. The data that conformed to the normal distribution were compared using the independent sample T-test, and the data that did not conform to the normal distribution was tested using the Wilcoxon rank-sum test. Graphing was done using GraphPad Prism 8. Statistical differences are expressed as follows: *P < 0.05; **P < 0.01; ***P < 0.001.

### Supplementary Information

Supplementary Information.

Supplementary Figures.

## Data Availability

The datasets generated and/or analysed during the current study are available in the manuscript.
