# Supplementary material for: Hepatoprotective effects of aspirin on diethylnitrosamine-induced hepatocellular carcinoma in rats by reducing inflammation levels and PD-L1 expression
Source: Sci Rep. 2023 Dec 4;13:21362. doi: 10.1038/s41598-023-48812-z (PMC10695938; doi:10.1038/s41598-023-48812-z)
Supplement: Supplementary file 1 — Supplementary Information. [file 41598_2023_48812_MOESM1_ESM.docx]

**Supplementary Information** Images of all replicate original blots of Figure 8 were included in Supplementary Figure 1. The blots were cut prior to hybridization with antibodies during blotting. We used red boxes to denote the regions of the original blots used in the main figures and used a red arrow to indicate the band position of the target protein: **(a).** Original blots of Figure 8a. **(b & c)**. Original blots of Figure 8b and c.
