# Supplementary figures and images for: Hepatoprotective effects of aspirin on diethylnitrosamine-induced hepatocellular carcinoma in rats by reducing inflammation levels and PD-L1 expression
Source: Sci Rep. 2023 Dec 4;13:21362. doi: 10.1038/s41598-023-48812-z (PMC10695938; doi:10.1038/s41598-023-48812-z)

**a**

$\beta$ -tubulin  
56 kDa

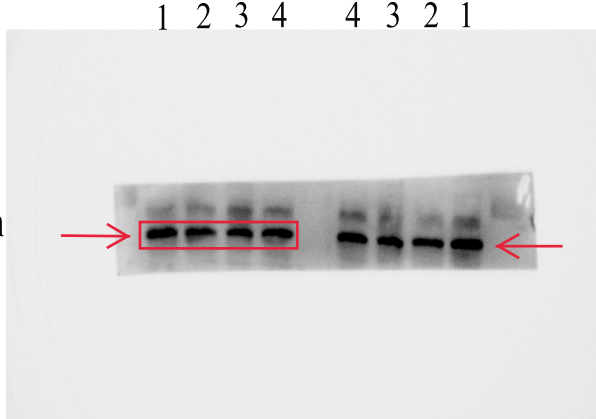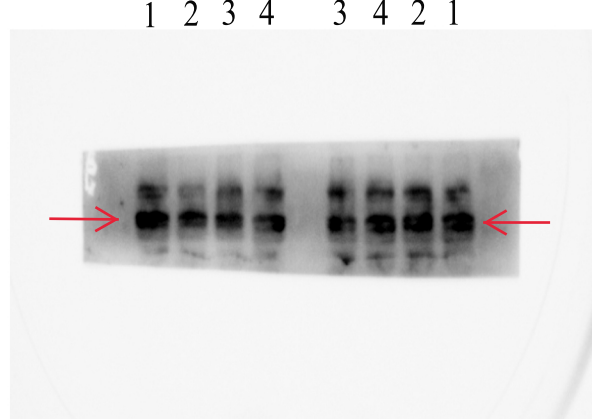

1 DEN  
2 DEN+ASA  
3 ASA  
4 Control

PD-L1  
36 kDa

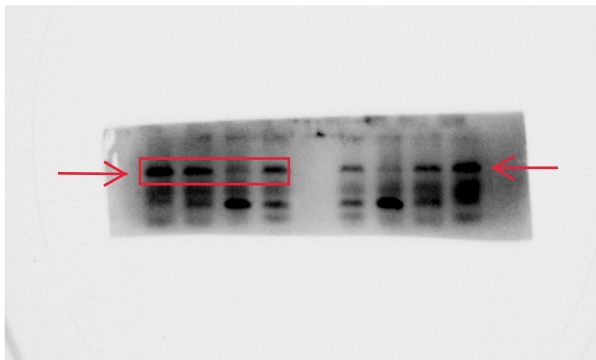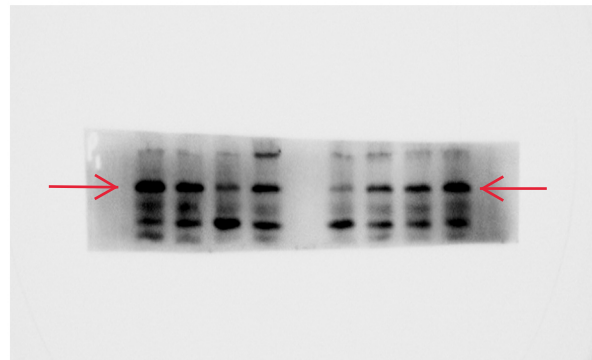

**b & c** **$\beta$ -actin**  
**42 kDa**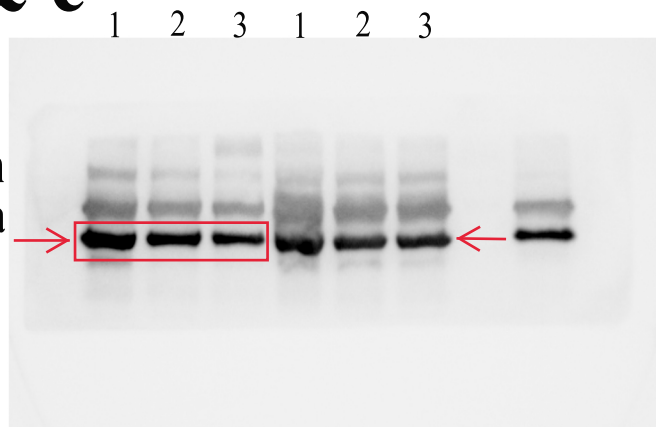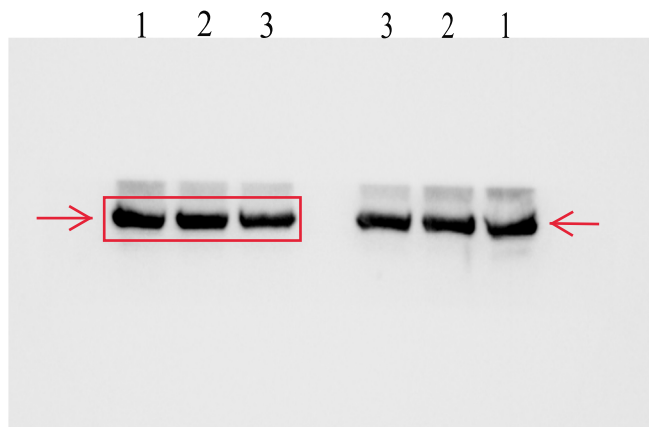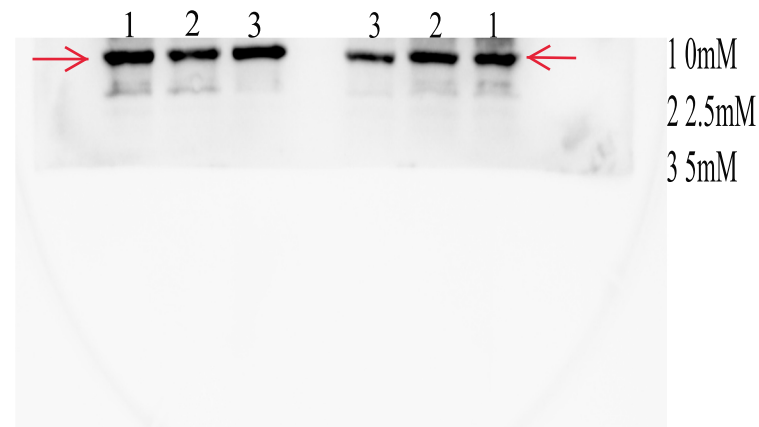**PD-L1**  
**36 kDa**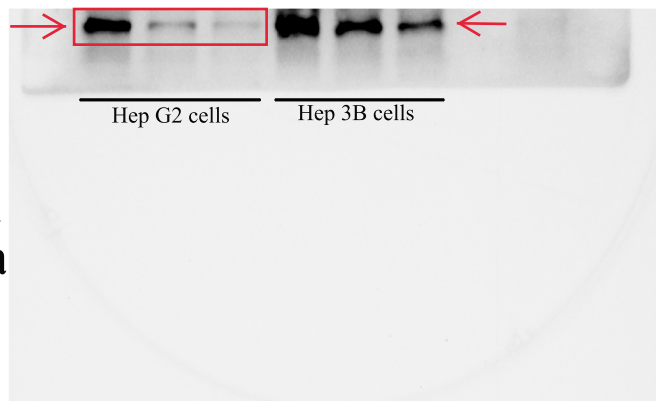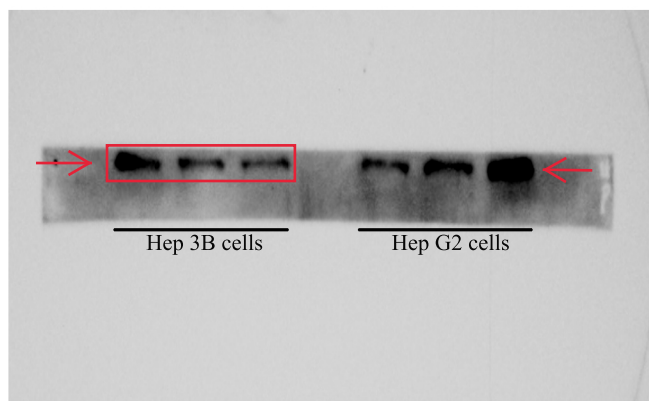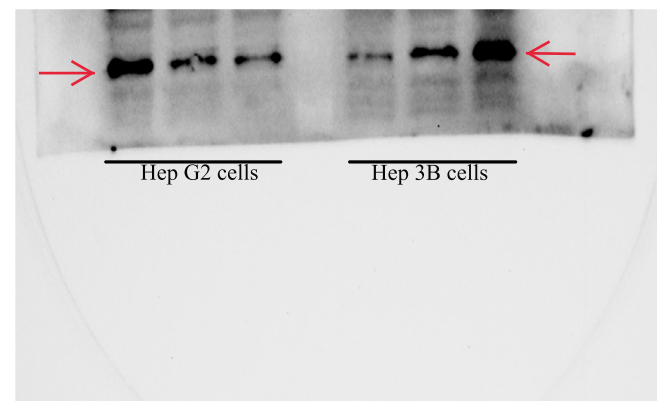

Supplement: Supplementary file 2 — Supplementary Figures. [file 41598_2023_48812_MOESM2_ESM.pdf]
